# Single VDGA-Based Mixed-Mode Electronically Tunable First-Order Universal Filter

**DOI:** 10.3390/s23052759

**Published:** 2023-03-02

**Authors:** Natchanai Roongmuanpha, Nutcha Likhitkitwoerakul, Masaaki Fukuhara, Worapong Tangsrirat

**Affiliations:** 1 School of Engineering, King Mongkut’s Institute of Technology Ladkrabang (KMITL), Bangkok 10520, Thailand; 2 Graduate School of Information and Telecommunication Engineering, Tokai University, 2-3-23, Takanawa, Minato, Tokyo 108-8619, Japan

**Keywords:** voltage differencing gain amplifier (VDGA), first-order filter, mixed-mode, dual-mode

## Abstract

This article presents a mixed-mode electronically tunable first-order universal filter configuration employing only one voltage differencing gain amplifier (VDGA), one capacitor, and one grounded resistor. With the appropriate selection of the input signals, the proposed circuit can realize all three first-order standard filter functions, namely low pass (LP), high pass (HP), and all pass (AP), in all four possible modes, including voltage mode (VM), trans-admittance mode (TAM), current mode (CM), and trans-impedance mode (TIM), from the same circuit structure. It also provides an electronic tuning of the pole frequency and the passband gain by varying transconductance values. Non-ideal and parasitic effect analyses of the proposed circuit were also carried out. PSPICE simulations and experimental findings have both confirmed the performance of the design. A number of simulations and experimental observations confirm the viability of the suggested configuration in practical applications.

## 1. Introduction

Continuous-time analog filter design is still a significant crucial and challenging topic of research. In recent years, the universal active filter configurations, which enable the simultaneous realization of multiple filtering functions namely lowpass (LP), highpass (HP), and allpass (AP) filters from the same topology, have received a lot of attention. The primary reasons for the widespread use of these filters are their applications in electronic sensors and instruments, control systems, and data communications. In particularly, the universal active filters play an important function as a circuit component in sensor applications such as biosensor systems, electrocardiogram (EKG) recording systems, phase sensitive detectors, etc. In practice, the high-order active filter design with an odd order also necessitates the use of first-order universal filters. As a result of this motivation, significant efforts have been devoted to designing first-order universal filters using a variety of modern analog active building blocks [1,2,3,4,5,6,7,8,9,10,11,12,13,14,15,16,17,18,19,20,21,22,23,24,25,26,27].

In [1], three different filter functions were implemented simultaneously in voltage-mode (VM) using two second-generation current conveyors (CCIIs), i.e., one CCII+ and one CCII−, two floating resistors, two grounded resistors, and one grounded capacitor. In the case of AP filter realization, the circuit must have an equal resistor condition in order to provide independent controllability of the natural frequency (*f_o_*). Using a single fully differential current conveyor (FDCCII), three resistors, one grounded capacitor, and three different first-order filter configurations with voltage input, voltage and current outputs are proposed in [2]. A versatile first-order current-mode (CM) universal filter employing two multiple-output CCIIs (MO-CCIIs), one resistor, and one capacitor is reported in [3]. The reported circuit employs a single grounded capacitor, which is suitable for integrated circuit (IC) implementation. It does not, however, provide electronic tuning of the *f_o_* parameter. The use of a single differential voltage current conveyor (DVCC) with two resistors and one grounded capacitor in realizing a VM first-order universal filter is presented in [4]. In the AP realization case, this circuit involves a lot of matching requirements. Again in [5], a DVCC-based VM first-order universal filter configuration is reported, employing two DVCCs, one grounded resistor and one grounded capacitor. The work of [6] describes a single multi-output operational transconductance amplifier (MO-OTA)-based first-order AP filter and amplitude equalization. However, the circuits given do not include all of the first-order generic filter functions. In [7], the CM first-order universal filter is implemented with only a single dual-X second generation multi-output current conveyor (DX-MOCCII) and four passive components. Despite the fact that experimental data were utilized to validate the filter’s practicability, it still lacked electronic adjustment of the *f_o_*. The earlier circuit in [8] reported a CM first-order filter design with low input and high output impedance utilizing two MO-CCIIs and all the three grounded passive elements. The element-matching limitations are imposed to implement all three first-order filter functions. The topology described in [9] details a single DVCC-based VM first-order universal filter constructed with one floating resistor and one grounded capacitor. By selecting appropriate input voltages, all three first-order filter functions can be obtained without any matching criteria. According to [10], a digitally programmable VM first-order universal filter based on a digitally controlled current conveyor (DPCCII) with three matched resistors and one grounded capacitor has been designed. As reported in [11], a CM multifunction first-order filter design with a single current differencing buffered amplifier (CDBA), two resistors, and one grounded capacitor was realized, which can be used to synthesize LP and HP filter responses simultaneously. Since the terminal n is not used in this structure, the full capacity of the CDBA device is not utilized. Furthermore, to construct the CM first-order universal filter in [12], two dual-output CCIIs (DO-CCIIs), a floating resistor, and a grounded capacitor were employed. No matching restriction was applied to realize LP, HP, and AP responses for this circuit. In [13], two inverting CCIIs (ICCIIs), one electronic MOS resistor, and a floating capacitor were used to realize both inverting and non-inverting CM first-order LP, HP, and AP functions from a single configuration. There are no critical passive element matching choices in the design. Although the first-order universal filter circuits in [14,15] only need a single active element and two passive grounded components, the internal construction of the device is rather sophisticated, requiring at least 40 MOS transistors along with compensating capacitor and resistor. Electronic control of these circuits is not possible. Two voltage subtractors, one floating resistor, and one grounded capacitor based on two different topologies of first-order VM filter functions were been proposed in [16]. Neither filters require any restrictions on passive component matching, but they cannot be electrically controlled. In [17], a single extra-X current controlled conveyor (EX-CCCII)-based first-order CM filter topology utilizing a single grounded capacitor is presented. This configuration offers low input and high output impedance and only generates three generic current filter functions simultaneously. No matching requirements are necessary for any of the three realized filter functions. The work in [18] reported an electronically controllable first-order universal filter with two operational transconductance amplifiers (OTAs), a grounded resistor, and a grounded capacitor that has ideal infinite input and output impedances. The circuit described therein only performs VM filter functions; for AP filters, the circuit necessitates matching constraints. Two different first-order filters are reported in [19], the first of which provides CM filter functions using a single multiple output dual-X current conveyor transconductance amplifier (MO-DXCCTA) and only one capacitor with three equal transconductances, and the second of which requires a MOSFET, a grounded capacitor, and one DXCCTA in order to realize three filter functions in transadmittance mode. A recently reported first-order generic CM filter circuit is based on a single modified DXCCTA [20]. The circuit that is being shown has low operating supply voltages, easy cascadability, and electronic tunability, however it is non-canonic in terms of the capacitors. The circuit reported in [21] is the resistorless realization of the CM universal filter and includes one differential difference dual-X second generation current conveyor (DD-DXCCII), four MOSFETs, and one grounded capacitor. However, to realize all three of the current transfer functions, it requires component-matching constraints. Based on two MO-CCIIs, one grounded resistor, and one grounded capacitor, the CM first-order universal filter configuration is introduced in [22]. This design can easily be cascaded and performs three current filter functions simultaneously without any matching requirements. Two plus-type inverting CCIIs (ICCII+s), one resistor, and one grounded capacitor were used to implement a first-order LP, HP, and AP filter in CM, as reported in [23]. The circuit offers electronic tunability and eliminates the need for restrictions on passive component matching. On the other hand, it is suggested in [24] to create a VM electrically tunable first-order universal filter utilizing a commercially available LT1228 IC. The circuit only employs one LT1228 IC, two floating resistors, and one floating capacitor, which is not ideal from the viewpoint of IC fabrication. In [25], five first-order universal filter designs using two current feedback operational amplifiers (CFOAs), three or four resistors, and a grounded capacitor are presented. Two of the five circuits require conditions for the realization of HP, while all five circuits require matching conditions for AP realization. The reported circuits offer filter capabilities in all four possible modes and include tunability features for gain and pole frequency, however, the filter parameters cannot be electronically controlled. The fully differential configuration of [26] employs a single multiple-output current differencing transconductance amplifier (MO-CDTA) and one capacitor to realize solely first-order LP, HP, and AP current responses within the same circuit design. Recently, a mixed-mode electronically tunable first-order universal filter structure was reported in [27]. To provide all three first-order generic filter functions in all four modes of operation, three OTAs and one grounded capacitor are required for its realization. The authors were inspired by the aforementioned critical review to continue working in this field and develop a novel minimum-component circuit for a first-order universal filter that operates in all four possible modes, namely VM, CM, trans-admittance-mode (TAM), and trans-impedance-mode (TIM).

Therefore, the primary objective of this work is to present a new mixed-mode first-order universal active filter design based on a single voltage differencing gain amplifier (VDGA), one resistor, and one capacitor that can derive LP, HP, and AP filter functions in all four possible modes by selecting the appropriate input voltages and currents. The passband gain and the pole frequency of the proposed filter can be electronically tuned using the transconductance gains of the VDGA. The performance of the proposed filter circuit was validated with PSPICE simulation results using TSMC 0.18-μm CMOS process technology. Experimental results using off-the-shelf IC type LM13600 OTAs are also included to support the theoretical propositions.

A thorough analysis of the previously reported first-order universal filter topologies was performed based on the aforementioned characteristics and a comparison was conducted with the proposed circuit, as given in Table 1. In summary, the following key contributions result from this work:

(1) The design of a novel first-order mixed-mode universal filter capable of realizing all three standard first-order filter functions and operating in all four operation modes with one active element and two passive elements;
(2) The realization of three filter responses in all four possible modes utilizing the same circuit configuration;
(3) The use of only grounded passive elements, except for HP, and AP filter functions in VM and TAM modes capable of absorbing parasitic elements;
(4) The proposed filter has an electronically adjustable pole frequency that has no effect on the passband gain of its responses;
(5) The practical implementation of the proposed filter using commercially available IC type is suggested;
(6) The performance of the proposed filter is proven through numerical simulations and hardware experiments.

## 2. Proposed Mixed-Mode First-Order Filter Configuration

**Figure 1:**
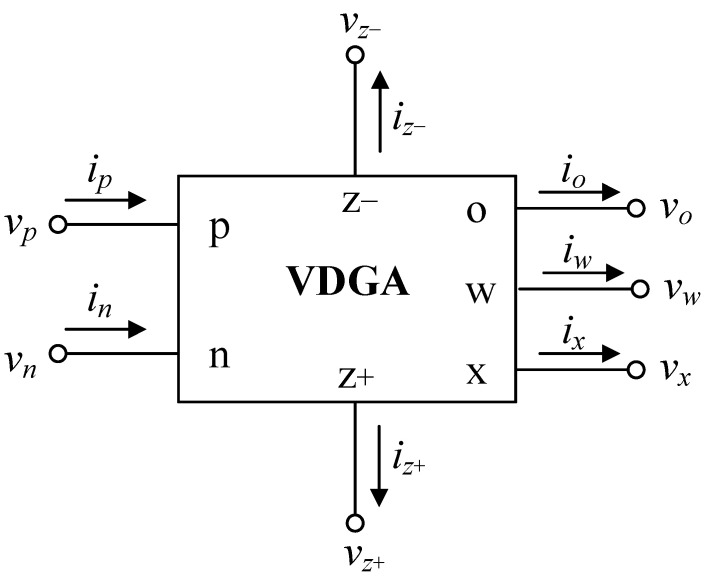
Symbol of VDGA.

The proposed first-order universal filter configuration is based on a single active element VDGA [28]. The VDGA device is a versatile and flexible active element with numerous solutions and applications [29,30,31,32]. A circuit symbol for VDGA is represented in Figure 1. Its terminal relationships are characterized below.

(1) $$\left[\begin{array}{c} i_{z+}\\ i_{z-}\\ i_{x}\\ \begin{array}{l} v_{w}\\ i_{o} \end{array} \end{array}\right]=\left[\begin{array}{cccc} g_{mA} & -g_{mA} & 0 & 0\\ -g_{mA} & g_{mA} & 0 & 0\\ 0 & 0 & -g_{mB} & 0\\ 0 & 0 & \beta & 0\\ 0 & 0 & 0 & g_{mC} \end{array}\right].\left[\begin{array}{c} v_{p}\\ v_{n}\\ v_{z+}\\ v_{w} \end{array}\right],$$

where *g_mk_* (*k* = *A*, *B*, *C*) and *β* are the transconductance gain and the transfer voltage gain of the VDGA, respectively.

**Figure 2:**
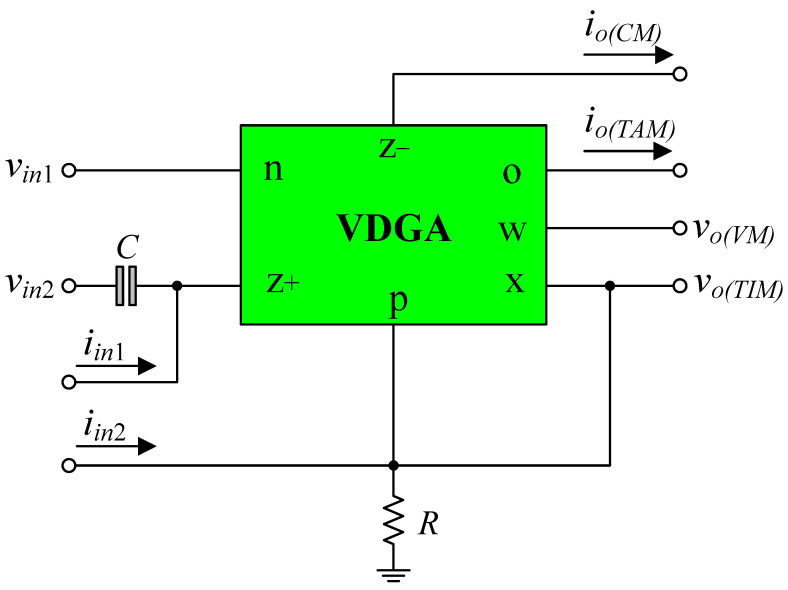
Proposed mixed-mode first-order universal filter.

Figure 2 shows the proposed first-order active universal filter that is comprised of one VDGA, one resistor, and one capacitor. The configuration can be utilized within the same circuit design to implement the mixed-mode first-order universal filter, which realizes LP, HP, and AP filter functions in VM, TAM, CM, and TIM, by appropriately selecting the input voltage and current signals, as specified below.

***Case I*:** If *i_in_*_1_ = *i_in_*_2_ = 0 (open circuited), the two-input one-output first-order universal filters in VM and TAM can be realized with the following transfer functions.

(a) VM filter
  (i) With *v_in_* = *v_in_*_1_ (input voltage) and *v_in_*_2_ = 0 (grounded), the following LP filter response is obtained from the *v_o(VM)_* terminal:
    (2) $$T_{VLP}(s)=\frac{v_{o(VM)}}{v_{in}}=\left(-\frac{1}{g_{mC}R}\right)T_{LP}(s).$$
  (ii) With *v_in_* = *v_in_*_2_ and *v_in_*_1_ = 0, the HP response is obtained as:
    (3) $$T_{VHP}(s)=\frac{v_{o(VM)}}{v_{in}}=\beta T_{HP}(s).$$
  (iii) With *v_in_* = *v_in_*_1_ = *v_in_*_2_ and *g_mB_R* = 1, the AP response is obtained as:
    (4) $$T_{VAP}(s)=\frac{v_{o(VM)}}{v_{in}}=\beta T_{AP}(s).$$

In the expressions above, the transfer functions *T_LP_*(*s*), *T_HP_*(*s*), and *T_AP_*(*s*) are written as follows.

(5) $$T_{LP}(s)=\frac{g_{mA}g_{mB}R}{D(s)},$$

(6) $$T_{HP}(s)=\frac{sC}{D(s)},$$

and

(7) $$T_{AP}(s)=\frac{sC-g_{mA}}{D(s)},$$

where

(8) $$D(s)=sC+g_{mA}g_{mB}R.$$

As shown in Equation (2), the LP first-order filter function circuit is realized by the proposed circuit with a passband gain of (−1/*g_mC_R*), as opposed to the others, which are expressed by Equations (3) and (4) and have a passband gain of *β*. It should be noted that the passband gains for three first-order filter responses can be electronically adjusted using the parameters *g_mC_* and *β*. Moreover, it was noticed from Equation (4) that a simple element requirement, *g_mB_R* = 1, is needed in the case of AP filter realization.

(b) TAM filter
  (iv) With *v_in_* = *v_in_*_1_ and *v_in_*_2_ = 0, the LP filter in TAM is obtained from the *i_o_*(*_TAM_*) terminal, as given by:
    (9) $$T_{YLP}(s)=\frac{i_{o(TAM)}}{v_{in}}=\left(-\frac{1}{R}\right)T_{LP}(s).$$
  (v) With *v_in_* = *v_in_*_2_ and *v_in_*_1_ = 0, the HP filter is realized as:
    (10) $$T_{YHP}(s)=\frac{i_{o(TAM)}}{v_{in}}=g_{mB}T_{HP}(s).$$
  (vi) With *v_in_* = *v_in_*_1_ = *v_in_*_2_ and *g_mB_R* = 1, the AP filter is realized as:
    (11) $$T_{YAP}(s)=\frac{i_{o(TAM)}}{v_{in}}=g_{mB}T_{AP}(s).$$

***Case II*:** If *v_in_*_1_ = *v_in_*_2_ = 0, three generic first-order filter functions in other two different operation modes, i.e., CM and TIM, may be derived, and their transfer functions can be given by the following.

(c) CM filter
  (vii) With *i_in_* = *i_in_*_1_ (input current) and *i_in_*_2_ = 0, the LP current filter response is obtained from the *i_o(CM)_* terminal:
    (12) $$T_{ILP}(s)=\frac{i_{o(CM)}}{i_{in}}=-T_{LP}(s).$$
  (viii) With *i_in_* = *i_in_*_2_ and *i_in_*_1_ = 0, the HP current response is obtained as:
    (13) $$T_{IHP}(s)=\frac{i_{o(CM)}}{i_{in}}=(g_{mA}R)T_{HP}(s).$$
  (ix) With *i_in_* = *i_in_*_1_ = *i_in_*_2_ and *g_mA_R* = 1, the AP current response is obtained as:
    (14) $$T_{IAP}(s)=\frac{i_{o(CM)}}{i_{in}}=(g_{mA}R)T_{AP}(s).$$

(d) TIM filter
  (x) With *i_in_* = *i_in_*_1_ and *i_in_*_2_ = 0, the following TIM LP filter is realized at the *v_o(TIM)_* output terminal:
    (15) $$T_{ZLP}(s)=\frac{v_{o(TIM)}}{i_{in}}=\left(-\frac{1}{g_{mA}}\right)T_{LP}(s).$$
  (xi) With *i_in_* = *i_in_*_2_ and *i_in_*_1_ = 0, the TIM HP filter is realized as:
    (16) $$T_{ZHP}(s)=\frac{v_{o(TIM)}}{i_{in}}=RT_{HP}(s).$$
  (xii) With *i_in_* = *i_in_*_1_ = *i_in_*_2_ and *g_mA_R* = 1, the TIM AP filter is realized as:
    (17) $$T_{ZAP}(s)=\frac{v_{o(TIM)}}{i_{in}}=RT_{AP}(s).$$

As is evident from all of the realized transfer functions given above, the circuit can consequently derive all three of the standard first-order filter functions, LP, HP, and AP, in all four operation modes using the same circuit topology. Thus, the proposed circuit of Figure 2 operates as a mixed-mode first-order universal filter with the pole frequency of

(18) $$\omega_{p}=2\pi f_{p}=\frac{g_{mA}g_{mB}R}{C}.$$

Obviously, the circuit has electronic tunability of the characteristic frequency *ω_p_* via *g_mA_* and *g_mB_*. Table 2 also summarizes the passband gains for the proposed filter operating in the four different modes. Based on the relationship between *ω_p_* in Equation (18) and the passband gain expressions in Table 2, it is possible to conclude that the passband gain of the LP, HP, and AP filters in VM can be tuned electronically and orthogonally by *g_mC_* without affecting *ω_p_*. The passband gains of HP and AP filters in TAM and CM can be electronically varied by *g_mB_* and *g_mA_*, respectively. In TIM, the LP passband gain is also electronically tunable via *g_mA_*.

## 3. Effect of Finite Tracking Errors

In a non-ideal case, the terminal relationships of VDGA taking into account the terminal tracking signal errors are specified as follows:

(19) $$\left[\begin{array}{c} i_{z+}\\ i_{z-}\\ i_{x}\\ \begin{array}{l} v_{w}\\ i_{o} \end{array} \end{array}\right]=\left[\begin{array}{cccc} \alpha_{A}g_{mA} & -\alpha_{A}g_{mA} & 0 & 0\\ -\alpha_{A}g_{mA} & \alpha_{A}g_{mA} & 0 & 0\\ 0 & 0 & -\alpha_{B}g_{mB} & 0\\ 0 & 0 & \delta \beta & 0\\ 0 & 0 & 0 & \alpha_{C}g_{mC} \end{array}\right].\left[\begin{array}{c} v_{p}\\ v_{n}\\ v_{z+}\\ v_{w} \end{array}\right].$$

In the above relationships, *α_k_* (*α_k_* = 1 − *ε_α_*) represents the non-ideal transconductance gain and *δ* (*δ* = 1 − *ε_δ_*) denotes the non-ideal voltage gain, both of which deviate from their ideal values due to the transfer signal errors *ε_α_* (|*ε_α_*| << 1) and *ε_δ_* (|*ε_δ_*| << 1). 

Considering the non-ideal characteristic of the VDGA in Equation (19), the various filter functions of the proposed circuit in Figure 2 for VM, TAM, CM, and TIM can be respectively expressed as follows:

(20) $$v_{o(VM)}=\frac{\delta \beta \left(sCv_{in2}-g_{mA}v_{in1}\right)}{D^{\prime }(s)},$$

(21) $$i_{o(TAM)}=\frac{\alpha_{C}\delta g_{mB}\left(sCv_{in2}-g_{mA}v_{in1}\right)}{D^{\prime }(s)},$$

(22) $$i_{o(CM)}=\frac{\alpha_{A}g_{mA}R\left(sCi_{in2}-\alpha_{B}g_{mB}i_{in1}\right)}{D^{\prime }(s)},$$

(23) $$v_{o(TIM)}=\frac{R\left(sCi_{in2}-\alpha_{B}g_{mB}i_{in1}\right)}{D^{\prime }(s)},$$

where

(24) $$D^{\prime }(s)=sC+\alpha_{A}\alpha_{B}g_{mA}g_{mB}R.$$

In view of the above expressions, the non-ideal pole frequency for the circuit becomes:

(25) $${\omega^{\prime }}_{p}=2\pi {f^{\prime }}_{p}=\frac{\alpha_{A}\alpha_{B}g_{mA}g_{mB}R}{C}.$$

Due to the non-ideal gains *α_A_* and *α_Β_*, the pole frequency of the proposed filter is altered slightly. However, since the values of *g_mA_* and *g_mB_* are electronically tunable, it is possible to compensate for the effects of the non-ideal gains *α_A_* and *α_Β_* by appropriately adjusting their values. Therefore, it is reasonable to conclude that the tracking errors of the VDGA parameters do not cause significant errors in the realized filter parameters. In addition to Equation (25), the factors *α_A_* and *α_Β_* depend primarily on the signal transfer errors of the VDGA. Consequently, the VDGA should be meticulously designed to prevent these errors. For typical tolerances obtained in contemporary integration processes, the introduced errors remain within acceptable parameters. 

## 4. Effect of Parasitic Elements

**Figure 3:**
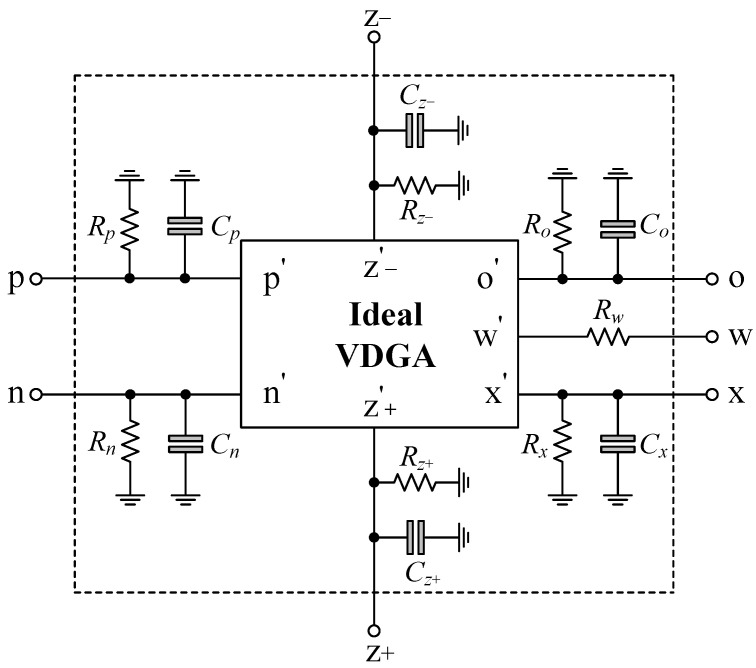
Equivalent circuit of the VDGA including various parasitic elements.

The non-ideal equivalent circuit of the VDGA, including various parasitic elements, is shown in Figure 3 [31,32]. These undesirable elements include parasitic resistances and capacitances, which look into the different VDGA terminals and affect the transfer functions of the proposed circuit. In consideration of the non-ideal behavior model of VDGA given in Figure 3, the characteristic equation of the proposed filter configuration in Figure 2 can be determined as follows:

(26) $$D^{\prime \prime }(s)=\frac{\left[R^{\prime }R_{z+}C^{\prime }\left(C_{p}+C_{x}\right)\right]}{\left[R^{\prime }\left(C_{p}+C_{x}\right)+R_{z+}C^{\prime }\right]}s^{2}+s+\left[\frac{\left(R^{\prime }R_{z+}g_{mA}g_{mB}+1\right)}{R^{\prime }\left(C_{p}+C_{x}\right)+R_{z+}C^{\prime }}\right],$$

where *R*′ = *R*//*R_p_*//*R_x_* and *C*′ = *C* + *C_z_*_+_. Choosing [*R*′(*C_p_* + *C_x_*) + *R_z_*_+_*C*′] >> [*R*′*R_z+_C*′(*C_p_* + *C_x_*)], then Equation (26) can be approximated as:

(27) $$D^{\prime \prime }(s)=s\left[R^{\prime }\left(C_{p}+C_{x}\right)+R_{z+}C^{\prime }\right]+\left(R^{\prime }R_{z+}g_{mA}g_{mB}+1\right).$$

As a result, the modified *ω″_p_* from Equation (27) may be written as:

(28) $${\omega^{\prime \prime }}_{p}=\frac{R^{\prime }R_{z+}g_{mA}g_{mB}+1}{R^{\prime }\left(C_{p}+C_{x}\right)+R_{z+}C^{\prime }}.$$

The parasitic impedances of the VDGA are observed to have an effect on the characteristic frequency *ω*″*_p_*. If we select *R* << (*R_p_*//*R_x_*), *R_z+_* and *C* >> *C_z_*_+_, (*C_p_* + *C_x_*), then we can suppose that *R* ≅ *R*′ and *C* ≅ *C*′, respectively, as a result the effect of the VDGA parasitics can be neglected. Additionally, this impact can also be mitigated by pre-distorting the values of *g_mA_* and *g_mB_*. 

## 5. Design and Simulation Verification

**Figure 4:**
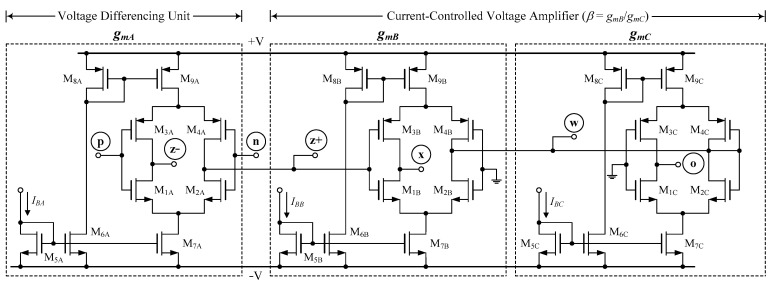
CMOS circuit of the VDGA used in simulation.

In this section, the PSPICE program is used to simulate the functionality of the proposed filter configuration shown in Figure 2. The CMOS circuit of Figure 4 [28,29,31,32] was used in simulation to implement the VDGA using 0.18-μm TSMC CMOS technology characteristics. The symmetrical DC supply voltages of ±0.9 V were used. Table 3 lists the transistor sizes utilized in the VDGA of Figure 4.

**Figure 5:**
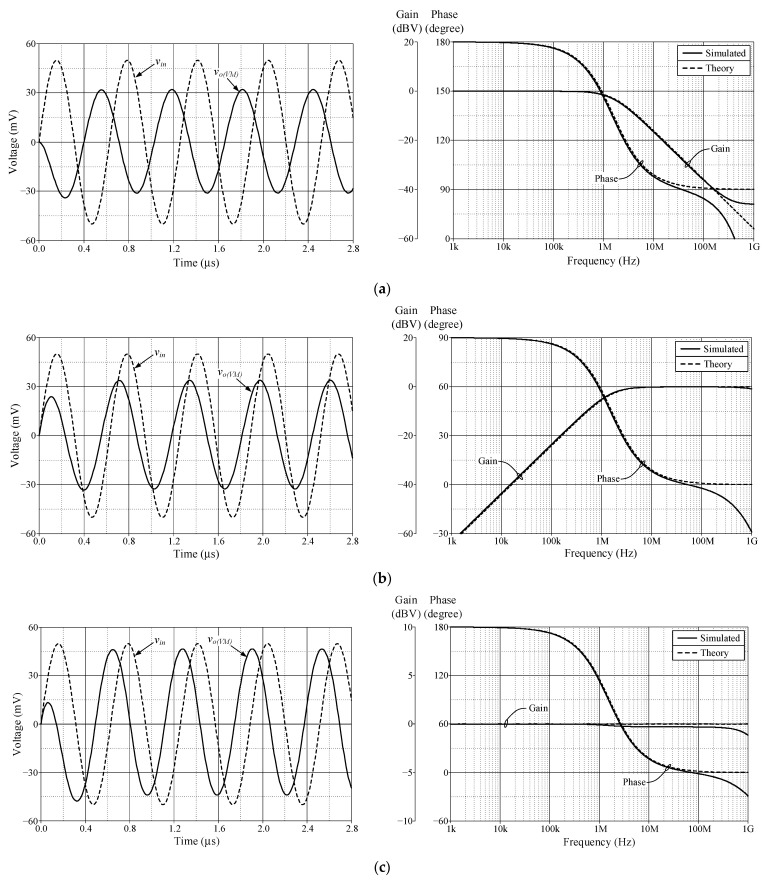
Simulated time and frequency responses of the proposed VM filter: (**a**) LP; (**b**) HP; and (**c**) AP.

**Figure 6:**
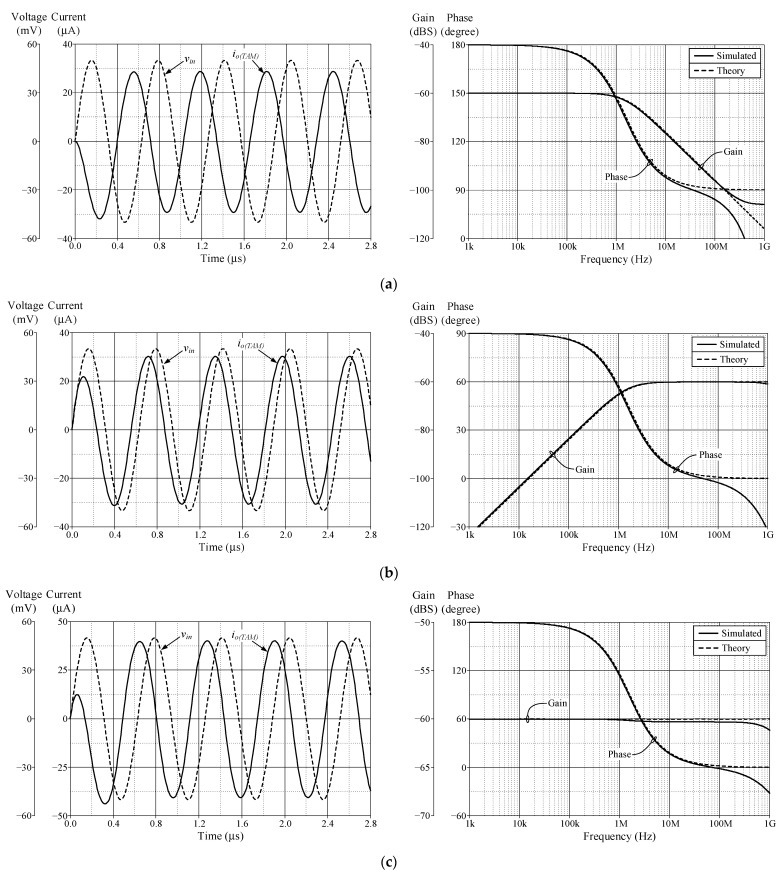
Simulated time and frequency responses of the proposed TAM filter: (**a**) LP; (**b**) HP; and (**c**) AP.

**Figure 7:**
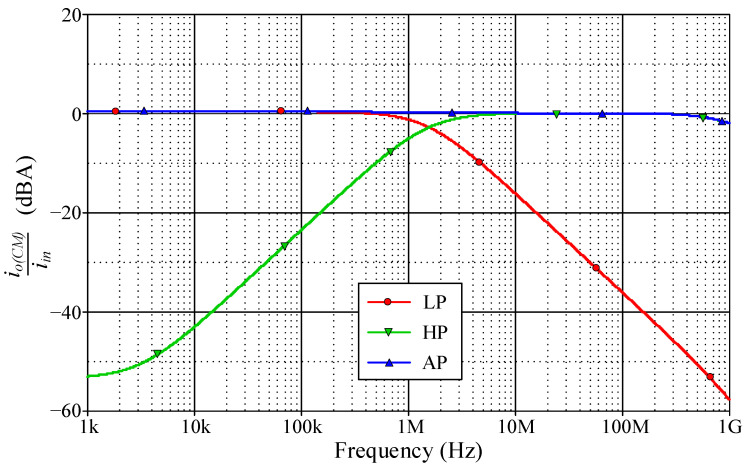
Simulated frequency responses of the proposed CM filter.

**Figure 8:**
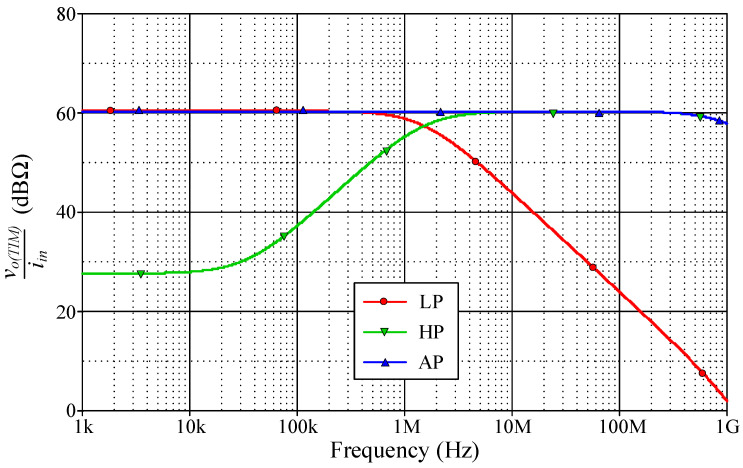
Simulated frequency responses of the proposed TIM filter.

As a design example, the proposed filter was realized for a pole frequency of 1.59 MHz. The designed component values for a given *f_p_* are *R* = 1 kΩ, *C* = 100 pF, and *g_mk_* = 1 mA/V (*I_Bk_* = 80 μA). Figure 5 and Figure 6 respectively show the simulated transient and frequency responses in VM and TAM, in comparison with the ideal responses. Similarly, the simulated and ideal frequency characteristics for the CM and TIM filters are given in Figure 7 and Figure 8, respectively. In transient response, the filter was fed a sinusoidal input signal with a peak amplitude of 50 mV at 1.59 MHz. The corresponding *f_p_* obtained from simulation results and their percentage errors from the theoretical values are given in Table 4. The simulated power dissipation of the circuit was determined to be 1.31 mW.

**Figure 9:**
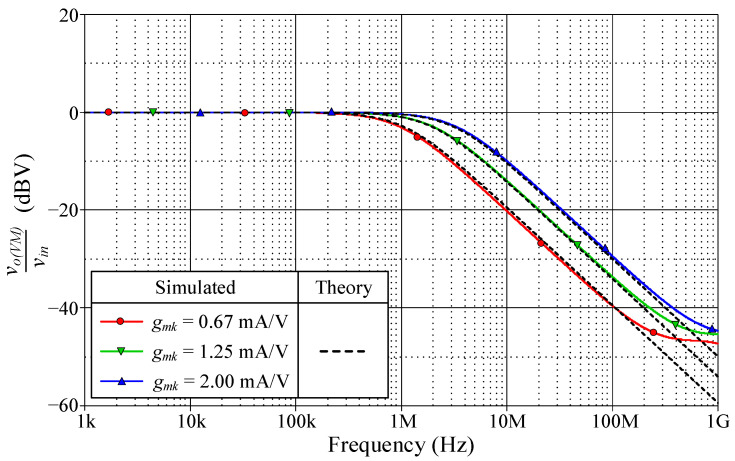
Tunability of *f_p_* of the proposed VM LP filter.

From Figure 5, Figure 6, Figure 7, Figure 8 and Figure 9, it is evident that the simulation results and theoretical values are in close agreement; however, there is a slight discrepancy at high frequencies due to the non-availability and limited frequency region of CMOS VDGA in an integrated form [28]. Note also that there is an external resistor *R*, as well as the parasitic resistances and capacitances connected from terminals p and x to ground. They become effective when operating at low frequencies. Therefore, the HP responses of the CM and TIM filters in Figure 7 and Figure 8 have non-ideal responses at low operating frequencies. This effect can be prevented by employing a smaller external resistor or operating the filter at a higher frequency.

Next, the electronic controllability of the pole frequency *f_p_* for the proposed LP filter in VM is demonstrated in Figure 9. The filter was designed to obtain *f_p_* = 1 MHz, 2 MHz, and 3.18 MHz by simply controlling *g_mk_* = 0.67 mA/V, 1.25 mA/V, and 2 mA/V. According to the simulation results, the corresponding *f_p_* were recorded at 0.98 MHz, 2.03 MHz, and 3.35 MHz, which are in error by 2%, 1.5%, and 5.34%, respectively.

**Figure 10:**
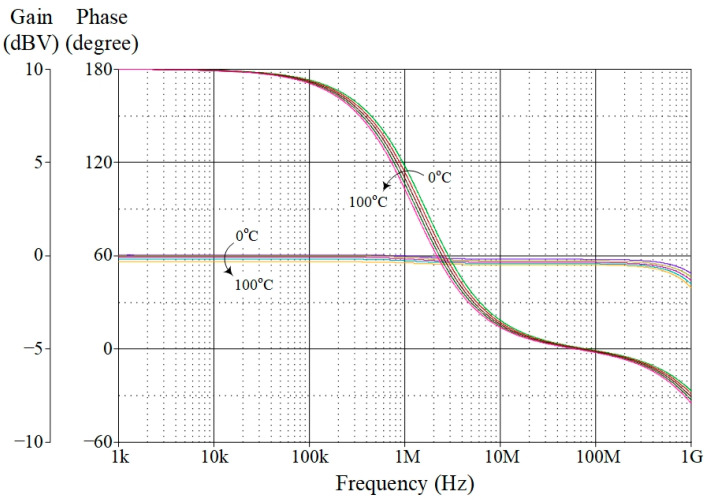
Simulated frequency characteristics of the proposed VM AP filter at temperatures of 0 °C, 25 °C, 50 °C, 75 °C, and 100 °C.

**Figure 11:**
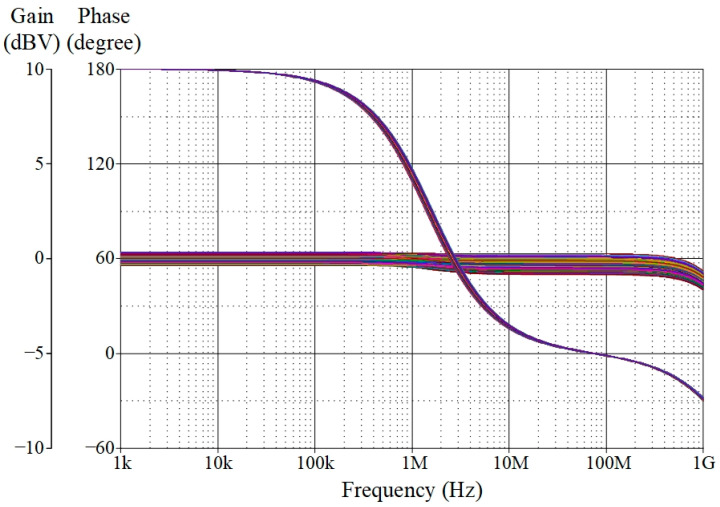
Monte-Carlo analysis results of the VM AP response at *f_p_* = 1.59 MHz.

The proposed VM AP filter was also simulated with changes in ambient temperature at 0 °C, 25 °C, 50 °C, 75 °C, and 100 °C. Figure 10 depicts the influence of temperature variation on the gain and phase responses of the filter. Based on the findings, the variances in gain and phase values for different temperatures are tabulated in Table 5, with theoretical gain and phase values of 0 dBV and 90°, respectively. Furthermore, a Monte Carlo (MC) statistical analysis of the VM AP filter at *f_p_* = 1.59 MHz was carried out with 5% tolerance of *g_mK_* and *C*. Figure 11 shows the MC simulation results of the filter’s gain and phase responses for 200 random runs with Gaussian distribution. The standard deviations of gain and phase were noted at ±0.21 dBV and ±2.18°, respectively.

## 6. Experiment-Based Validation

**Figure 12:**
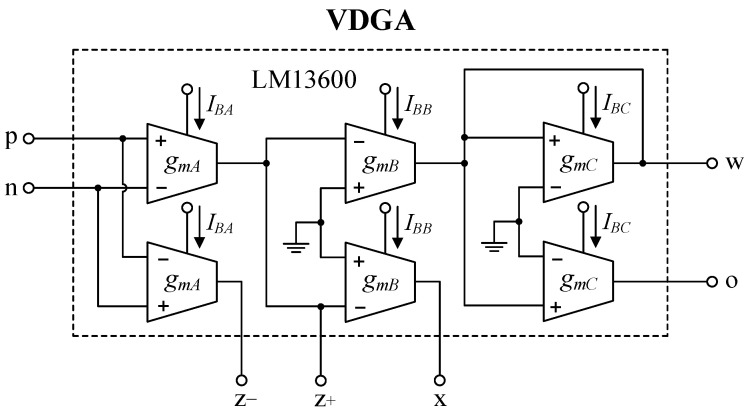
VDGA realization in experimental measurements using off-the-shelf available IC-type LM13600s.

The proposed circuit in Figure 2 was also validated experimentally to confirm the theoretical assumptions. The VDGA was implemented in practical measurements using readily available IC-type LM13600 dual-OTAs [33], as shown schematically in Figure 12. DC supply voltages of ±5 V were used to bias the OTAs. For experimental verification, the proposed filter circuit operating in all four modes was designed for the theoretical *f_p_* of 234 kHz with *R* = 1 kΩ, *C* = 680 pF, and *g_mk_* = 1 mA/V (*I_Bk_* = 50 μA). To obtain the current signal measurements, voltage-to-current and current-to-voltage converter circuits with IC CFOA AD844s and a converting resistor of 1 kΩ were utilized as described in [32].

**Figure 13:**
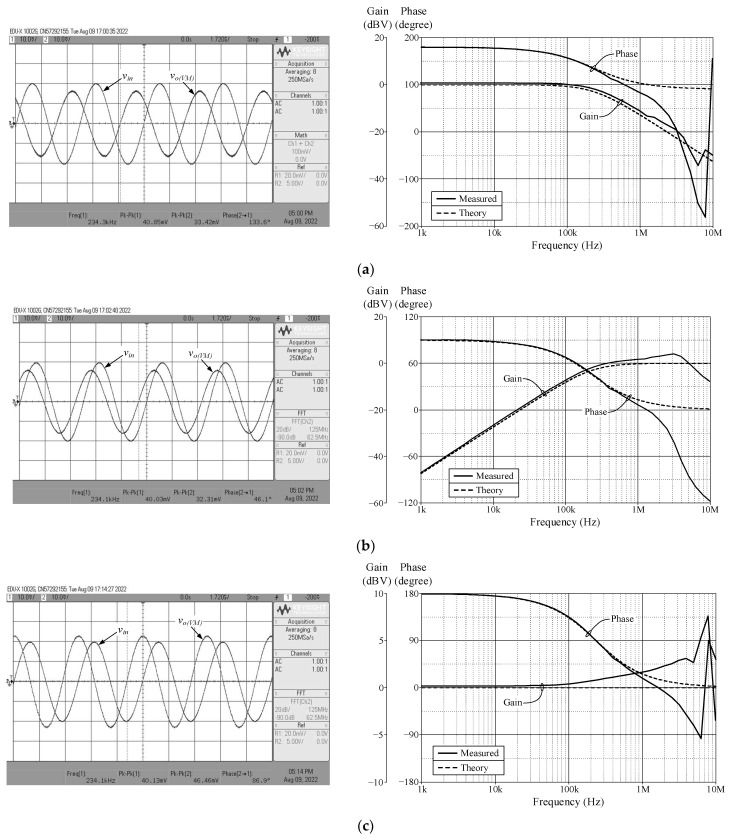
Measured time and frequency responses of the proposed VM filter: (**a**) LP; (**b**) HP; and (**c**) AP.

**Figure 14:**
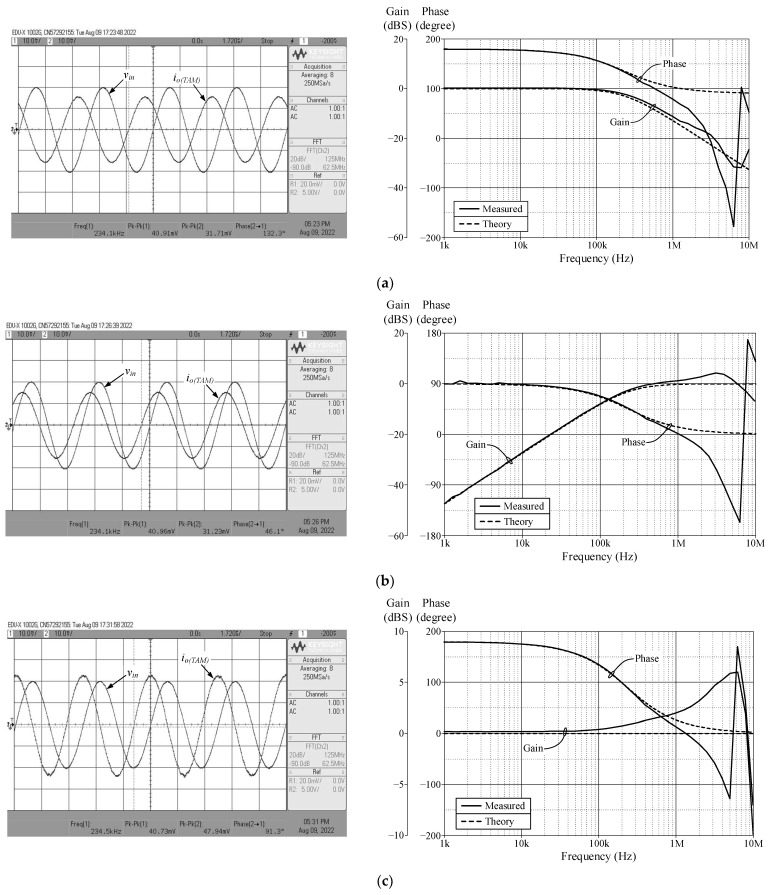
Measured time and frequency responses of the proposed TAM filter: (**a**) LP; (**b**) HP; and (**c**) AP.

**Figure 15:**
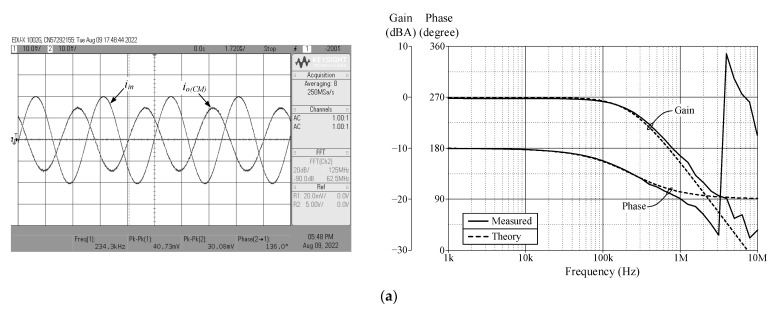

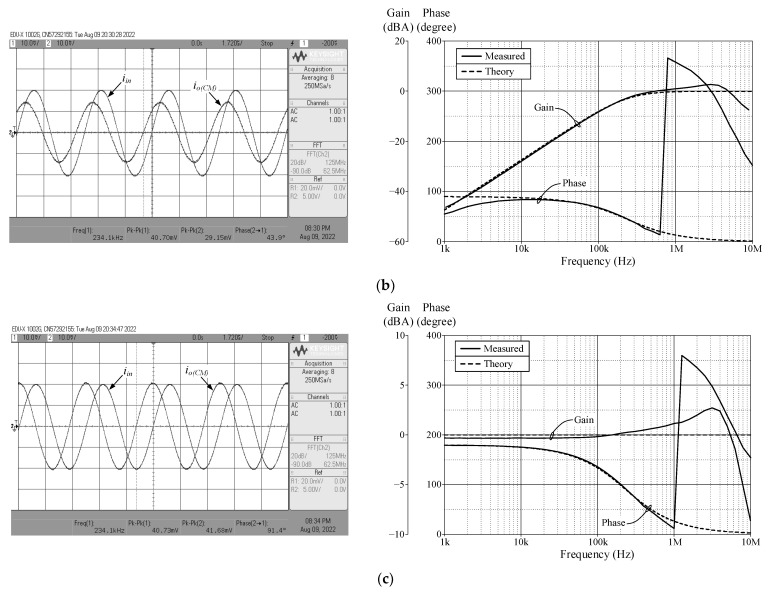
Measured time and frequency responses of the proposed CM filter: (**a**) LP; (**b**) HP; and (**c**) AP.

**Figure 16:**
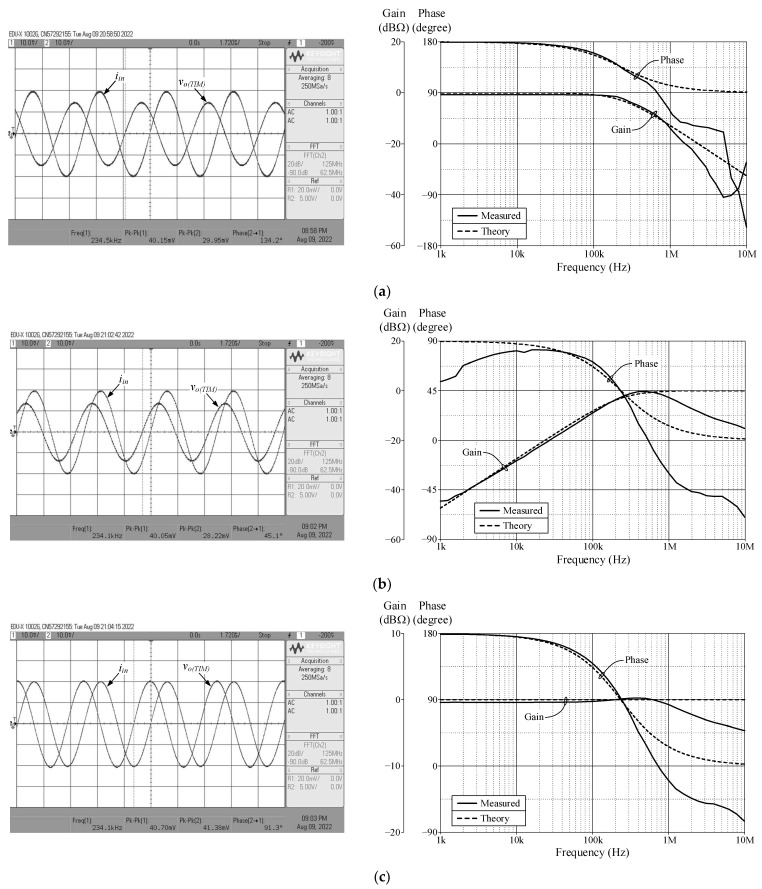
Measured time and frequency responses of the proposed TIM filter: (**a**) LP; (**b**) HP; and (**c**) AP.

**Figure 17:**
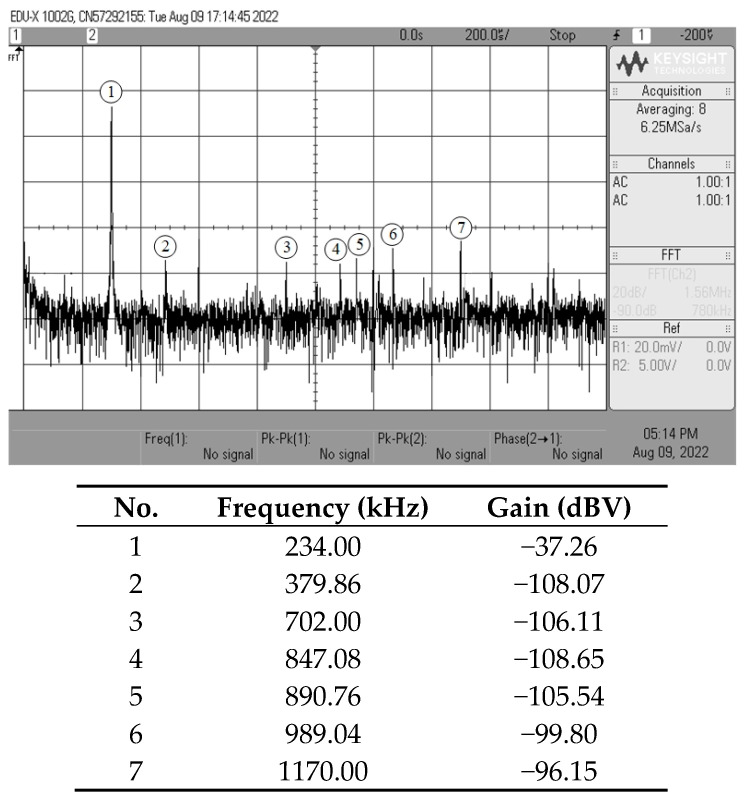
Experimentally observed frequency spectrum of *v_o(VM)_* of the AP filter in VM.

**Figure 18:**
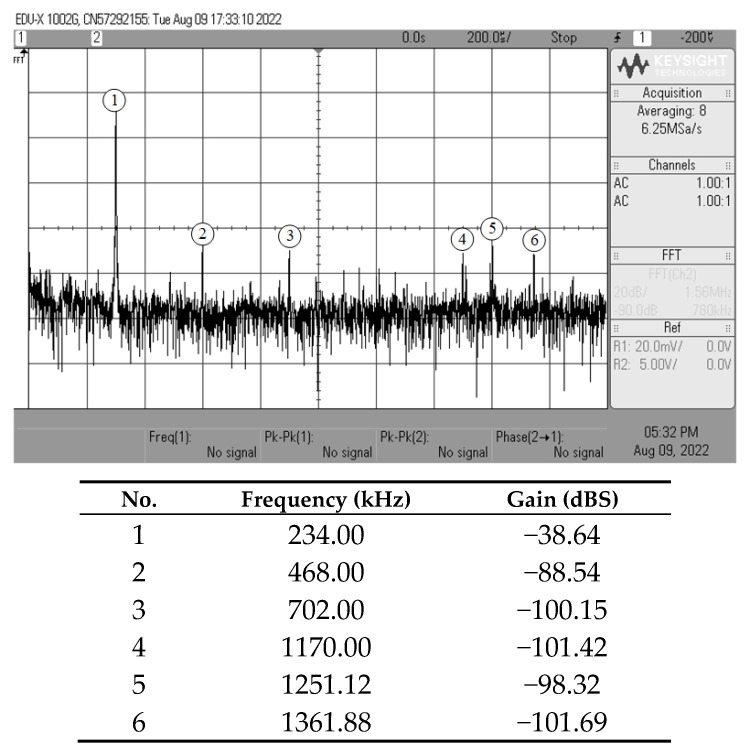
Experimentally observed frequency spectrum of *i_o(TAM)_* of the AP filter in TAM.

**Figure 19:**
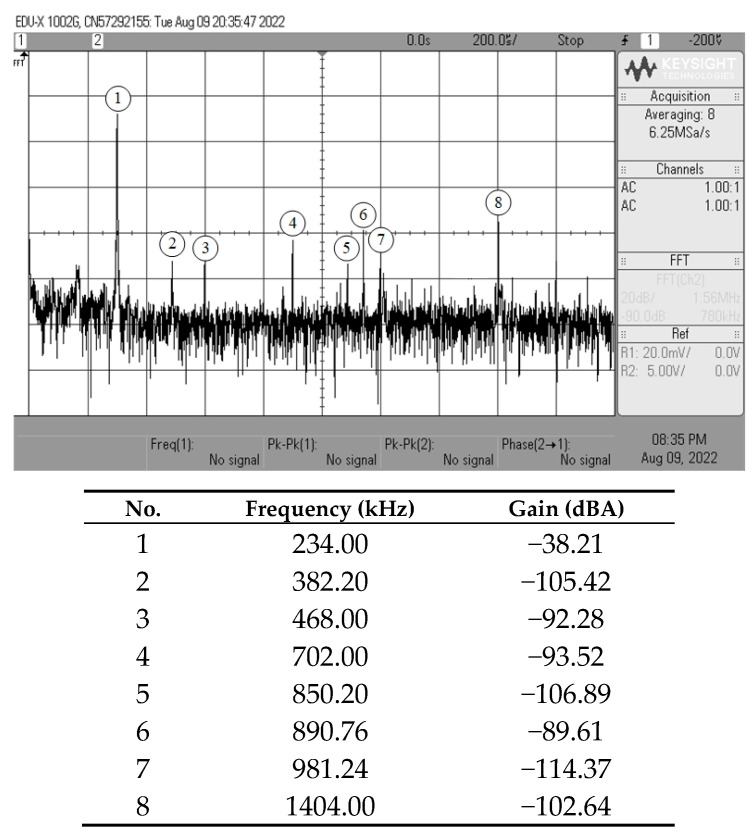
Experimentally observed frequency spectrum of *i_o(CM)_* of the AP filter in CM.

**Figure 20:**
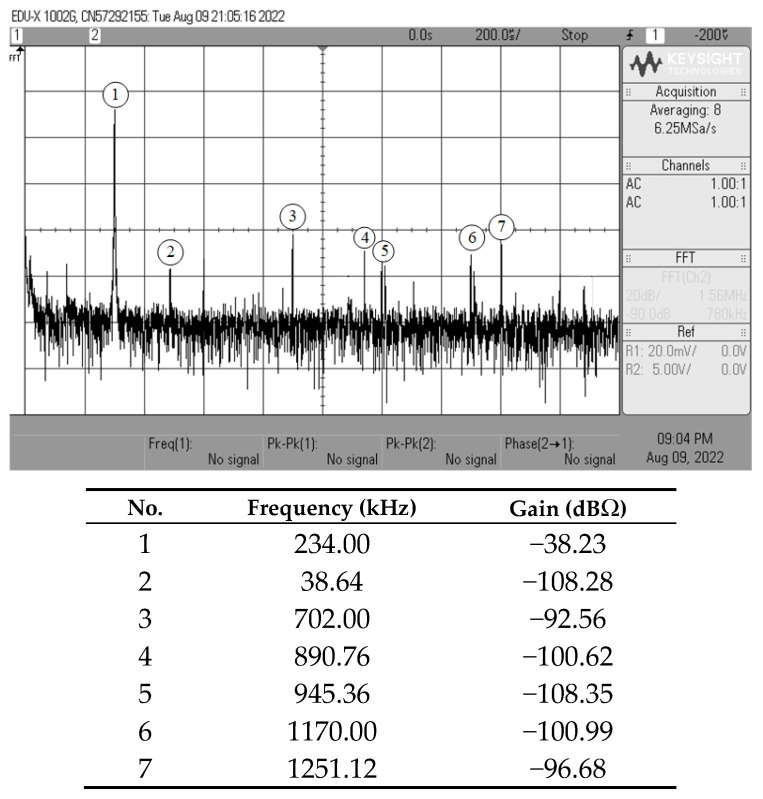
Experimentally observed frequency spectrum of *v_o(TIM)_* of the AP filter in TIM.

Figure 13, Figure 14, Figure 15 and Figure 16 show the experimentally observed waveforms of transient and frequency responses for each filter function in all four different modes. The input voltage (*v_in_*) and the input current (*i_in_*) for transient measurements were adjusted to 20 mV (peak) and 20 μA (peak), respectively, with a frequency of 234 kHz. Regarding all of the experimental results, the measured *f_p_* for each filter and the corresponding percentage deviations are recorded in Table 6. Figure 17, Figure 18, Figure 19 and Figure 20 also show the measured frequency spectrums of the AP filter output for each of the four modes. As a result of measurements, total harmonic distortion (THD) values were determined to be 0.15%, 0.36%, 0.38%, and 0.25% for VM, TAM, CM, and TIM, respectively.

The experimental results presented in Figure 13, Figure 14, Figure 15 and Figure 16 reveal that, despite the measured results being for signals in the kilohertz range, the proposed filter is capable of operating satisfactorily at much higher frequencies. It is also noted that the experimentally observed gain and phase responses are not ideal at high frequencies. Deviations in the gain and phase frequency responses can be attributed to the parasitics of the IC LM13600 used to implement the VDGA. More specifically, the 2 MHz gain bandwidth product of the IC LM 13600 [33] would degrade the high operating frequency. If a dedicated CMOS VDGA becomes available, this effect should no longer be an issue. 

## 7. Application to a Dual-Mode Quadrature Oscillator

**Figure 21:**
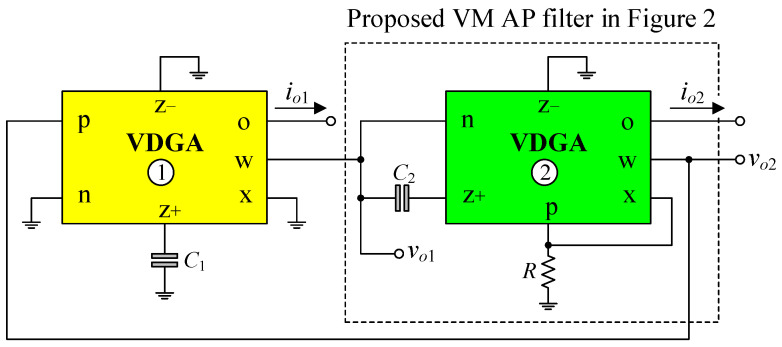
Dual-mode quadrature oscillator implemented from the proposed VM AP filter circuit.

As shown in Figure 21, the dual-mode quadrature oscillator (DM-QO), which provides both voltage and current quadrature outputs (*v_o_*_1_, *v_o_*_2_, *i_o_*_1_ and *i_o_*_2_), is derived from the proposed first-order voltage-mode AP filter. The first block is a VDGA-based dual-output lossless integrator, and the second one is the proposed VM AP filter circuit in Figure 2. If all the transconductances of both VDGAs are identical, such that *g_m_* = *g_mk_*, and *C* = *C*_1_ = *C*_2_, then the oscillation condition (OC) and the oscillation frequency (*f_o_*) of the DM-QO are derived as:

(29) $$\mathrm{OC}:g_{m}=\frac{1}{R},$$

and

(30) $$f_{o}=\frac{g_{m}\beta }{2\pi C}.$$

Additionally, the mathematical expressions for the voltages and currents at the quadrature outputs are, respectively,

(31) $$v_{o2}=jk_{1}v_{o1},$$

and

(32) $$i_{o2}=jk_{2}i_{o1},$$

where *k*_1_ = (2*πfC*/*g_m_β*) and *k*_2_ = (2*πfC*/*g_m_*). In accordance with Equations (31) and (32), the output voltages and currents have a phase difference of 90° in their respective waveforms. At the oscillation frequency (*f* = *f_o_*), both the coefficients *k*_1_ and *k*_2_ are made equal to unity. As a result, the DM-QO in Figure 21 will produce output voltages and currents with equal signal amplitudes that are in quadrature.

**Figure 22:**
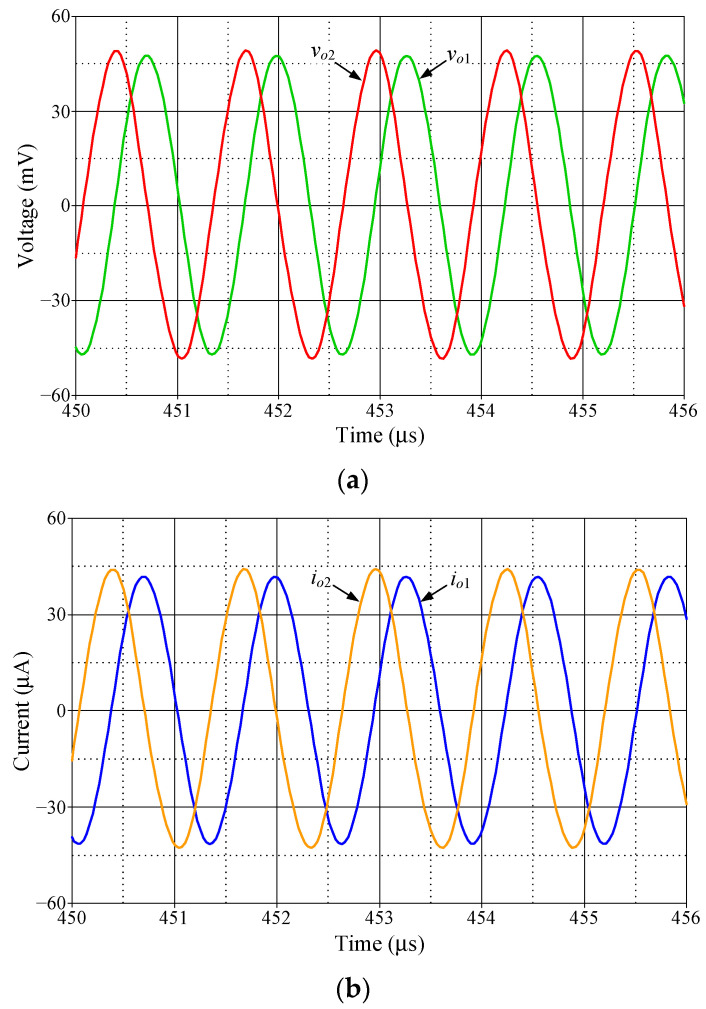
Simulated output waveforms for the DM-QO in Figure 21: (**a**) *v_o_*_1_ and *v_o_*_2_; (**b**) *i_o_*_1_ and *i_o_*_2_.

To demonstrate the performance of the DM-QO in Figure 21, the simulation was done using the CMOS VDGA of Figure 4. The DM-QO was designed to oscillate at *f_o_* = 1.59 MHz. The designed values of active and passive components were taken as: *R* = 1 kΩ, *C* = *C*_1_ = *C*_2_ = 100 pF, and *g_m_* = *g_mk_* = 1 mA/V. The simulated transient waveforms of output voltages and currents for the oscillator are depicted in Figure 22. The phase relationships of *v_o_*_1_-*v_o_*_2_ and *i_o_*_1_-*i_o_*_2_ were simulated to be 86.91° and 85.72°, which correspond to deviations of 3.43% and 4.75%, respectively. The percentage THDs of the simulated waveforms of voltages (*v_o_*_1_ and *v_o_*_2_) and currents (*i_o_*_1_ and *i_o_*_2_) were found to be: 4.03%, 5.46%, 5.17%, and 5.41%, respectively.

## 8. Conclusions

In this work, a single VDGA-based electronically tunable mixed-mode first-order universal filter is proposed, employing only one capacitor and one grounded resistor. All three general first-order filter functions−low pass, high pass, and all pass−can be realized by the proposed circuit in each of the four operational modes−VM, TAM, CM, and TIM. The pole frequency and the passband gain of the realized filter are capable of electronic tuning through the adjustment of the transconductance gains of the VDGA. An analysis of the non-ideal performance of the proposed circuit was examined, and the results were also discussed in comparison to the ideal analysis. The practical viability of the circuit was verified using both PSPICE simulation results and experimental measurements. Moreover, the dual-mode quadrature oscillator that can provide both quadrature output voltages and currents simultaneously was designed and simulated as an application example. The design of higher-order mixed-mode universal filters and mixed-mode multiphase sinusoidal oscillators will become the focus of future work.

## Figures and Tables

**Table 1:** Comparison among the earlier reported first-order universal filters [1,2,3,4,5,6,7,8,9,10,11,12,13,14,15,16,17,18,19,20,21,22,23,24,25,26,27] and the proposed circuit.

Ref.	Number of Active Element	Number of Passive Element	Available in Four Posible Modes	Filter Function Realized				Electronics Tunable	Passband Gain Tunable	*f_p_* (Hz)	Technology	Supply Voltages (V)	Power Consumption (W)	Technology	Supply Voltages (V)
				VM	CM	TAM	TIM								
[1]	CCII+ = 1, CCII− = 1	R = 4, C = 1	no	all three	--	--	--	no	VM: LP	200 k	AD844	N/A	N/A	--	--
[2]	FDCCII = 1	R = 3, C = 1	no	all three	--	Figure 3 and Figure 5: HP Figure 4: AP	--	no	TAM: HP, AP	79.6 k	--	--	--	--	--
[3]	MO-CCII = 2	R = 1, C = 1	no	--	all three	--	--	no	no	358 k	MIETEC 0.5 μm	±2.5, −1.79	N/A	--	--
[4]	DVCC = 1	R = 2, C = 1	no	all three	--	--	--	no	no	1.59 M	TSMC 0.35 μm	±1.65, −0.25, +0.5	N/A	--	--
[5]	DVCC = 2	R = 1, C = 1	no	all three	--	--	--	no	no	397 k	TSMC 0.18 μm	±1.25, ±0.53	N/A	--	--
[6]	OTA = 1, MO-OTA = 1	C = 1	no	--	AP	--	--	yes	no	1 M	MOSIS 0.5 μm	±2	N/A	--	--
[7]	DX-MOCCII = 1	R = 2, C = 2	no	--	all three	--	--	no	CM: LP, HP	1.59 M	TSMC 0.25 μm	±1.25	N/A	AD844	±10
[8]	CCII = 2	R = 2, C = 1	no	--	all three	--	--	no	no	1.32 M	TSMC 0.18 μm	±1.25, −0.6	N/A	--	--
[9]	DVCC = 1	R = 1, C = 1	no	all three	--	--	--	no	no	1.59 M	TSMC 0.18 μm	±0.9, −0.1, −0.36	N/A	--	--
[10]	DPCCII = 1	R = 3, C = 1	no	all three	--	--	--	no	no	7.96 k	TSMC 0.25 μm	±0.75	N/A	--	--
[11]	CDBA = 1	R = 2, C = 1	no	--	all three	--	--	no	CM: LP	159 k	AD844	±5	N/A	--	--
[12]	DO-CCII = 2	R = 1, C = 1	no	--	all three	--	--	no	no	6.37 M	IBM 0.13 μm	±0.75	4.8 m	--	--
[13]	ICCII = 2	R_MOS_ = 1, C = 1	no	--	all three	--	--	yes	no	2.6 M	IBM 0.13 μm	±0.75, +0.37	2.75 m	--	--
[14]	DX-MOCCII = 1	R = 1, C = 1	no	--	all three	--	--	no	no	7.96 M	TSMC 0.25 μm	±1.25, −0.3	N/A	--	--
[15]	FTFN = 2	R = 2, C = 2, switch = 1	no	all three	--	--	--	no	no	1 M	AMS 0.35 μm	±1.65	18.2m	AD844	N/A
[16]	Subtractor = 2	R = 1, C = 1	no	all three	--	--	--	no	no	6.37 M	IBM 0.13 μm	±0.75, +0.24	1.77 m	AD844	±6
[17]	EX-CCCII = 1	C = 1	no	--	all three	--	--	yes	no	3.93 M	TSMC 0.25 μm	±1.25	4.24 m	--	--
[18]	OTA = 2	R = 1, C = 1	no	all three	--	--	--	yes	VM: HP	8.05 k	TSMC 0.18 μm	±0.4	47.2 μ	LM13700	±5
[19]	MO-DXCCTA = 1	Figure 1: C = 1, Figure 2: R_MOS_ = 1, C = 1	no	--	Figure 1: all three	Figure 2: all three	--	yes	TAM: LP, HP, AP	11.7 M	TSMC 0.18 μm	±1.25, +0.42	1.47 m	AD844, LM13700	±10
[20]	DXCCTA = 1	C = 2	no	--	all three	--	--	yes	no	10 M	TSMC 0.18 μm	±1.25, +0.42	1.75 m	AD844, LM13700	±5
[21]	DD-DXCCII = 1	R_MOS_ = 3, C = 1	no	--	all three	--	--	yes	CM: LP, HP	3 M	TSMC 0.18 μm	±1.25, −0.6	2 m	--	--
[22]	Figure 2: MO-CCII = 2	R = 1, C = 1	no	--	all three	--	--	no	no	15.55 M	TSMC 0.18 μm	±1.25, +0.6	3.71 m	--	--
	Figure 9: DDCC = 2	R = 1, C = 1	no	all three	--	--	--	no	no	15.8 M			3.71 m	--	--
[23]	Figure 2: ICCII+ = 2	R = 1, C = 1	no	--	all three	--	--	no	no	7.96 M	IBM 0.13 μm	±0.75, +0.23	3.29 m	AD844	±9
[24]	LT1228 = 1	R = 2, C = 1	no	all three	--	--	--	yes	VM: LP, HP	90 k	LT1228	±5	5.76 m	LT1228	±5
[25]	CFOA = 2	R = 3–4, C = 1	no	all three	--	--	--	no	VM: LP, HP, AP	159 k	--	--	--	AD844	±12
[26]	MO-CDTA = 1	C = 1	no	--	all three	--	--	yes	no	1.59 M	TSMC 0.13 μm	±1, −0.56	2.5 m	AD844, LM13700	±10
[27]	OTA = 3	C = 1	yes	all three	all three	all three	all three	yes	VM: HP, CM: LP, TAM: LP, HP, AP, TIM: LP, HP, AP	159 k	TSMC 0.18 μm	±0.9, −0.785	N/A	LM13700	±15
Proposed circuit	VDGA = 1	R = 1, C = 1	yes	all three	all three	all three	all three	yes	VM: LP, HP, AP, CM: HP, AP, TAM: LP, HP, AP, TIM: LP, HP, AP	1.59 M	TSMC 0.18 μm	±0.9	1.31 m	LM13600	±5

Abbreviations: R = resistor, C = capacitor, N/A = not available, “--” = not realized, RMOS = MOS-based electronic resistor, FTFN = four terminal floating nullor.

**Table 2:** Passband gains of the proposed mixed-mode first-order filter in Figure 2.

Mode of Operation	LP	HP	AP
VM	(−1/*g_mC_R*)	*β*	*β*
TAM	(−1/*R*)	*g_mB_*	*g_mB_*
CM	−1	*g_mA_R*	*g_mA_R*
TIM	(−1/*g_mA_*)	*R*	*R*

**Table 3:** Transistor dimensions of VDGA in Figure 4.

Transistors	*W* (μm)	*L* (μm)
M_1*k*_–M_2*k*_	23.5	0.18
M_3*k*_–M_4*k*_	30	0.18
M_5*k*_–M_7*k*_	5	0.18
M_8*k*_–M_9*k*_	5.5	0.18

**Table 4:** Simulated *f_p_* and percentage errors of the proposed filter in Figure 2.

	VM		TAM		CM		TIM	
	*f_p_* (MHz)	Error (%)	*f_p_* (MHz)	Error (%)	*f_p_* (MHz)	Error (%)	*f_p_* (MHz)	Error (%)
LP	1.51	5.03	1.49	6.29	1.49	6.29	1.49	6.29
HP	1.50	5.66	1.49	6.29	1.50	5.66	1.45	8.81
AP	1.52	4.40	1.52	4.4	1.52	4.40	1.49	6.29

**Table 5:** Gain and phase values of the proposed VM AP filter at *f_p_* for different temperatures.

	Temperature				
	0 °C	25 °C	50 °C	75 °C	100 °C
Gain (dBV)	−0.06	−0.14	−0.22	−0.32	−0.42
Phase (degree)	92.34	87.94	83.83	80.04	76.57

**Table 6:** Measured *f_p_* and percentage errors of the proposed filter in Figure 2.

	VM		TAM		CM		TIM	
	*f_p_* (kHz)	Error (%)	*f_p_* (kHz)	Error (%)	*f_p_* (kHz)	Error (%)	*f_p_* (kHz)	Error (%)
LP	228.04	2.54	231.31	1.14	240.59	2.81	231.12	1.23
HP	251.18	7.34	237.98	1.70	241.54	3.22	228.04	2.54
AP	231.31	1.14	230.74	1.33	237.72	1.58	237.31	1.41

## Data Availability

The data supporting the results presented in this work are available on request from the authors.
